# The effectiveness of cold-knife conization (CKC) for post-menopausal women with cervical high-grade squamous intraepithelial lesion: a retrospective study

**DOI:** 10.1186/s12893-021-01238-8

**Published:** 2021-05-12

**Authors:** Xiao Li, Meihua Liu, Yurou Ji, Pengpeng Qu

**Affiliations:** 1 Clinical College of Central Gynecology and Obstetrics, Tianjin Medical University, Heping, Tianjin, 300070 People’s Republic of China; 2 Department of Gynecological Oncology, Tianjin Central Hospital of Gynecology Obstetrics, Tianjin Key Laboratory of Human Development and Reproductive Regulation, 156 Nankai Third Road, Nankai, Tianjin, 300100 People’s Republic of China

**Keywords:** Colposcopy, Conization, High-grade squamous intraepithelial lesion, Hysterectomy, Post-menopause

## Abstract

**Background:**

The effectiveness of surgery of high-grade squamous intraepithelial lesion in post-menopausal women needs to be investigated. This study evaluated the clinical significance of cold-knife conization in the diagnosis and surgery of cervical high-grade squamous intraepithelial lesions in post-menopausal women.

**Methods:**

We conducted a retrospective analysis of post- and pre-menopausal patients with high-grade squamous intraepithelial lesion. All patients received cold-knife conization as the primary therapy.

**Results:**

The satisfactory rate of colposcopy was significantly lower in the post-menopausal group than in the pre-menopausal group (38.33 vs. 71.25%; *χ*^2^ = 36.202, *P* < 0.001). The overall positive margin rate of cold-knife conization (25.83 vs 12.50%; *χ*^2^ = 10.106, *P* = 0.001) and rate of positive endocervical cone margins (16.67 vs. 4.58%; *χ*^2^ = 14.843, *P* < 0.001) were significantly higher in the post-menopausal group. Moreover, 49 post- and 60 pre-menopausal women underwent subsequent surgical treatment (40.83 vs. 25.00%). Residual rate of positive and negative margins in patients before and after menopause was significantly different (*χ*^2^ = 5.711, *P* = 0.017; *χ*^2^ = 12.726, *P* < 0.001, respectively). The recurrence rate in post-menopausal women remained 3.85%.

**Conclusions:**

Cold-knife conization can be performed as a primary procedure for diagnosis and surgery of post-menopausal patients with high-grade squamous intraepithelial lesions. Sufficient deep excisions are necessary to avoid positive endocervical margins, which can reduce the residual and recurrence of postoperative lesions.

## Introduction

Cervical squamous intraepithelial lesions (CSIL) are a group of cervical lesions that are closely associated with invasive cervical cancer. Cervical squamous intraepithelial lesions/Cervical intraepithelial lesions (CIN) can be divided into two categories: low-grade squamous intraepithelial lesion (LSIL/CIN1) and high-grade squamous intraepithelial lesion (HSIL/CIN2,3). The immediate treatment of HSIL (CIN2,3) is usually necessary, as the spontaneous regression rates at these stages are low (32%-43%); if such disease is left untreated, the risk of progression to invasive cancer is substantially increased by 5–22% [1, 2].Cervical conization, as a conservative surgical approach to treat HSIL, includes cold-knife conization (CKC), loop electrosurgical excision procedure (LEEP), and laser conization. These procedures are suitable particularly for women who are young or who desire to preserve their fertility. Compared to pre-menopausal women, post-menopausal women have quite different physiological characteristics, such as a decline in estrogen levels and cervical atrophy. Therefore, the effectiveness of surgery of HSIL in post-menopausal women needs to be investigated. In this study, we aimed to compare post-menopausal and pre-menopausal patients with HSIL who had undergone CKC and then evaluate the clinical significance of CKC in the diagnosis and surgery of HSIL in post-menopausal women.

## Materials and methods

A retrospective analysis of 360 patients with HSIL diagnosed by colposcopy-directed biopsy and histological analysis was performed from March 2014 to August 2015 at our hospital. The study sample included 120 post-menopausal and 240 pre-menopausal patients as controls (a ratio of 1:2). Patients with a minimum of 12 months of spontaneous amenorrhea were considered post-menopausal.This study conforms to the provisions of the Declaration of Helsinki (as revised in Tokyo 2004).

Information about personal history (age, gravidity, parity, menopausal age, symptoms, ThinPrep cytologic test (TCT), high-risk human papillomavirus (HR-HPV) test, colposcopic evaluation, final pathological result) was available for every patient. These factors were compared between the post- and pre-menopausal groups. Colposcopy was considered satisfactory if the full extent of the cervical lesion and the entire or partial transformation zone were visible. The examination was classified as unsatisfactory if the entire cervical lesion and transformation zone were not visualized.

All patients underwent CKC as the primary therapy. Cervical conization is separated into three types, requiring removal of the complete transformation zone and part of the cervical canal above the squamocolumnar junction( SCJ). Normally, type I resection is used for type 1 transformation zone, and the resection depth is 7–10 mm. Type II resection is used for type 2 transformation zone, and the resection depth is 10–15 mm. Type III resection is used for type 3 transformation zone, the resection depth is 15–25 mm. The type of cervical conization should be selected based on the results of the colposcopy evaluation and ultrasound cervical status. Type III cervical conization is generally preferred in postmenopausal women.All specimens were diagnosed by experienced gynecological pathologists at the Department of Pathology at our hospital. When there was inconsistency of pathological grade between colposcopy-directed biopsy, conization, and hysterectomy, the highest grade was the final diagnosis. No lesion or inflammatory change on conization margins was considered negative. Margins were reported as positive if HSIL or cancer existed at or near (≤ 1 mm) the resection surface. If postoperative pathology of cervical invasive carcinoma, positive margins, extensive lesions, residual lesions, or uterine or ovarian lesions were identified, then further surgery was required. Patients with stage IA1 cervical cancer without fertility requirements underwent extrafascial hysterectomy, and patients with extensive lesions and residual lesions also underwent this surgery.Patients with stage IA2-IB1 cervical cancer underwent modified radical or radical hysterectomy and lymphadenectomy. Positive margins after cervical conization were classified as HSIL or cancer. Patients whose resection margins were HSIL preferred extrafascial hysterectomy or secondary conization. Patients whose resection margins were cancer and did not preserve fertility underwent radical hysterectomy and pelvic lymphadenectomy.Cytology analysis and HR-HPV DNA test were required during follow-up,and those with abnormalities were referred for colposcopy, cervical biopsy and endocervical curettage(ECC).Recurrence was defined as histopathological HSIL during follow-up.

## Statistical analysis

Statistical evaluation of data was performed using SPSS software version 17.0 (SPSS, Chicago, IL). Enumeration data were compared by chi-square test. *P* < 0.05 was considered statistically significant.

## Results

### Preoperative clinical features

In the post-menopausal group, the mean age of the patients was 54 (range = 45–65) years. The average gravidity and parity were 3 and 1.4, respectively. The mean age of menopause was 50 (range = 39–58) years. 50 patients had clinical symptoms: abnormal vaginal bleeding (n = 31, 25.83%), leukorrhagia (n = 8, 6.67%), abnormal vaginal bleeding and leukorrhagia (n = 7, 5.83%), or lumbosacral pain (n = 4, 3.33%). In the pre-menopausal group, the mean age of the patients was 45 (range = 19–55) years. The average gravidity and parity were 2.8 and 1.2, respectively. Clinical symptoms occurred in 104 patients: abnormal vaginal bleeding (n = 53, 22.08%), leukorrhagia (n = 31, 12.92%), abnormal vaginal bleeding and leukorrhagia (n = 16, 6.67%), or lumbosacral pain (n = 4, 1.67%). Over half of all patients did not experience any symptoms (Table 1).

**Table 1:** Preoperative clinical features of the 2 groups

	Clinical symptoms				No clinical symptoms n (%)
	Abnormal vaginal bleeding n (%)	Leukorrhagia n (%)	Abnormal vaginal bleeding and leukorrhagia n (%)	Lumbosacral pain n (%)	
Post-menopausal patients (n = 120)	31 (25.83%)	8 (6.67%)	7 (5.83%)	4 (3.33%)	70 (58.33%)
Pre-menopausal patients (n = 240)	53 (22.08%)	31 (12.92%)	16 (6.67%)	4 (1.67%)	136 (56.67%)

### Preoperative laboratory examinations

In total, 110 of the 120 post-menopausal patients and 227 of the 240 pre-menopausal patients had TCT. Cytological analysis results revealed a 30.91% (34/110) diagnosis consistency between the cytology and biopsy histology in the post-menopausal women and 32.16% (73/227) in the pre-menopausal women. No significant difference was observed between the 2 groups (χ2 = 0.053, P = 0.817). Moreover, 112 post-menopausal patients and 224 pre-menopausal patients underwent HPV testing. The results demonstrated that the positive rate of HR-HPV was 91.07% (102/112) in the post-menopausal women and 94.20% (211/224) in the pre-menopausal women. There was no significant difference between the 2 groups (*χ*^2^ = 1.143, *P* = 0.285; Tables 2 and 3).

**Table 2:** Results of cervical cytology and consistency between the cytology and biopsy histology in post- and pre-menopausal patients

	Cytology								Consistency between the cytology and biopsy histology, n (%)
	NILM	ASCUS	ASC-H	LSIL	HSIL	SCC	AGC	Undone	
Post-menopausal Patients (n = 120)	18	37	14	5	34	1	1	10	34 (30.91%)
Pre-menopausal Patients (n = 240)	17	88	22	23	73	0	4	13	73 (32.61%)
χ^2^ P-value									0.053 0.817

*AGC* atypical glandular cells, *ASCUS* atypical squamous cell of undetermined significance, *ASC-H* atypical squamous cell-cannot exclude HIS, *HSIL* high-grade squamous intraepithelial lesions, *LSIL* low-grade squamous intraepithelial lesions, *NILM* negative for intraepithelial lesion or malignancy, *SCC* squamous cell carcinoma, *Undone* no ThinPrep cytologic test

**Table 3:** Results of HR-HPV in post- and pre-menopausal patients

	HR-HPV		
	Positive, n (%)	Negative, n (%)	Undone, n
Post-menopausal patients (n = 120)	102 (91.07%)	10 (8.93%)	8
Pre-menopausal patients (n = 240)	211 (94.20%)	13 (5.80%)	16
*χ*^2^ *P*-value	1.143 0.285		

### Satisfactory rate of colposcopy and consistency of pathological results between biopsy and conization of the 2 groups

All patients underwent colposcopy, of which 46 (38.33%) and 171 (71.25%) cases in the post-menopausal and pre-menopausal group, respectively, were satisfied with colposcopy. The satisfactory rate of colposcopy was significantly lower in the post- than in the pre-menopausal group (*χ*^2^ = 36.202, *P* < 0.001). Taking the histological diagnosis of CIN by conization as the golden standard, the consistency of colposcopy-directed biopsy had no significant difference in the post- and pre-menopausal group (75.83 vs. 83.75%, *χ*^2^ = 3.273, *P* = 0.07). The upgrading between biopsy and conization was significantly higher in the post- than in the pre-menopausal group (11.67 vs. 5.42%, *χ*^2^ = 4.505, *P* = 0.03) (Tables 4 and 5).

**Table 4:** Histology results after CKC

Histology	Post-menopausal Patients (n = 120)	Pre-menopausal patients (n = 240)
No lesion	13	21
LSIL	2	5
HSIL	91	201
Cancer	14	13

**Table 5:** Satisfactory rate of colposcopy before and after menopause and the consistency of pathological result between biopsy and conization

	Post-menopausal patients, n (%)	Pre-menopausal patients, n (%)	*χ*^2^	*P*-value
Satisfactory rate of colposcopy	46 (38.33%)	171 (71.25%)	36.202	*P* < 0.001
Consistency between biopsy and conization	91 (75.83%)	201 (83.75%)	3.273	0.070
Upgrading between biopsy and conization	14 (11.67%)	13 (5.42%)	4.505	0.034

### Positive margin rate and additional surgery

The overall positive margin rate of CKC was 25.83% in the post-menopausal group, which was significantly higher than the rate (12.50%) in the pre-menopausal group (*χ*^2^ = 10.106, *P* = 0.001). Among them, the rate of positive endocervical cone margins in the post-menopausal group was significantly higher than the rate of positive margins in the pre-menopausal group (16.67 vs. 4.58%, *χ*^2^ = 14.843, *P* < 0.001). The positive ectocervical margin rates were 9.16% and 7.92% in the post- and pre-menopausal groups, respectively. There was no significant difference between the 2 groups (*χ*^2^ = 0.164, *P* = 0.686).

A total of 49 post-menopausal patients and 60 pre-menopausal patients underwent subsequent surgical treatment (40.83 vs. 25.00%). In the post-menopausal group, 14 patients were diagnosed as having invasive cervical cancer following conization treatment, including 9 stage IA1, 1 stage IA2, and 4 stage IB1. 9 of these patients had positive margins. Among 31 patients with positive margins, 26 cases were HSIL with resection margins, including 22 cases of CIN3 and 4 cases of stage IA1 cervical cancer. 2 cases of CIN3 refused to reoperation, and the rest underwent extrafascial hysterectomy. 5 cases of resection margins were cancer, including 4 cases of stage IB1 and 1 case of stage IA2 cervical cancer, all underwent radical hysterectomy and pelvic lymphadenectomy.Moreover, 5 cases of cervical cancer of stage IA1 with negative margins, 6 cases with extensive lesions, and 2 cases with residual lesions underwent extrafascial hysterectomy. 2 patients underwent hysterectomy because of leiomyoma, and 5 patients underwent extrafascial hysterectomy because of concern about disease progression or absence of follow-up conditions. In the pre-menopausal group, 13 patients were diagnosed with invasive cervical cancer (5 cases of positive margins), including 8 stage IA1, 2 stage IA2, and 3 stage IB1. Among the 30 patients with positive margins, 26 cases were HSIL with resection margins, including 25 cases of CIN3 and 1 case of stage IA1 cervical cancer. 7 cases of CIN3 refused to reoperation, 18 cases underwent extrafascial hysterectomy, and 1 case of CIN3 underwent secondary CKC. 4 cases of resection margin were cancer, including 3 cases of stage IB1 and 1 case of stage IA2 cervical cancer, all underwent radical hysterectomy and pelvic lymphadenectomy. 2 patients with stage IA1 who desired further reproduction did not undergo subsequent surgical treatment. The remaining 5 cases of stage IA1 and 1 case of stage IA2 cervical cancer with negative margins underwent extrafascial hysterectomy, modified radical hysterectomy and pelvic lymphadenectomy.Moreover, 14 cases with extensive lesions and 8 patients because of concern about disease progression or absence of follow-up conditions underwent extrafascial hysterectomy. 9 patients underwent hysterectomy because of amalgamative uterine or ovarian disease. Among them, 6 cases of leiomyoma, 2 cases of adenomyosis, 1 case of ovarian endometriosis. The patients who had not undergone further surgery had regular cytology, HPV and colposcopy examinations.Histologic analysis of the second specimen (reconization or hysterectomy) showed residual disease in 14 of 49 post-menopausal patients and in 20 of 60 pre-menopausal patients. There was no significant difference between the 2 groups (28.57 vs. 33.33%, *χ*^2^ = 0.285, *P* = 0.593). Moreover, 49 post-menopausal patients underwent a second surgical treatment, of which 29 had positive margins and 20 had negative margins. The incidence rates of residual disease in patients with positive and negative margins after CKC were 41.38 and 10.00%, respectively. Among 60 pre-menopausal patients who underwent subsequent surgical treatment, 23 patients had positive margins and 37 patients had negative margins. The rates of residual disease of positive and negative margins were 60.87and 16.22%, respectively. The residual rate of positive and negative margins in patients before and after menopause were significantly different (*χ*^2^ = 5.711, *P* = 0.017; *χ*^2^ = 12.726, *P* < 0.001). The rate of residual disease had no significant difference between the post- and pre-menopausal patients with positive margins (*χ*^2^ = 1.949, *P* = 0.163; Table 6).

**Table 6:** Positive margin rate and residual disease in post- and pre-menopausal patients

	Post-menopausal patients, n (%)	Pre-menopausal patients, n (%)	*χ*^2^	*P*-value
Overall positive margin rate	31 (25.83%)	30 (12.50%)	10.106	0.001
Positive endocervical margin rate	20 (16.67%)	11 (4.58%)	14.843	*P* < 0.001
Positive ectocervical margin rate	11 (9.16%)	19 (7.92%)	0.164	0.686
Residual disease	14 (28.57%)	20 (33.33%)	0.285	0.593
Residual disease of positive margin	12 (41.38%)^a^	14 (60.87%)^b^	1.949	0.163
Residual disease of negative margin	2 (10.00%)	6 (16.22%)		

^a^Residual disease of positive margin vs. negative margin in the post-menopausal patients: *χ*^2^ = 5.711, *P* = 0.017

^b^Residual disease of positive margin vs. negative margin in the pre-menopausal patients: *χ*^2^ = 12.726, *P* < 0.001

### Follow-up results

The average follow-up period was 25 (range = 6–43) months. 16 of 120 and 27 of 240 patients in the post- and pre-menopausal groups, respectively, were lost to follow-up. 17 patients had cytological abnormalities during follow-up in the post-menopausal group, including 12 cases of squamous cells of undetermined significance (ASCUS), 2 cases of atypical squamous cells, excluding HSIL (ASC-H), 1 case of LSIL, 1 case of HSIL, and 1 case of atypical glandular cells. 14 patients underwent colposcopy-directed biopsy. The remaining 3 patients whose cytology revealed ASCUS and HPV-negative did not undergo colposcopy, and the cytology turned negative after a few months.Biopsy pathologic diagnosis revealed 4 cases of HSIL, 2 cases of LSIL, and 1 case of VaINI. There was a special case after extrafascial hysterectomy in which cytology was normal but colposcopy-directed biopsy and pathology revealed vaginal squamous cell carcinoma. 4 cases of HSIL were considered recurrence, which were diagnosed 4–17 months after primary therapy, and the recurrence rate was 3.85%. These patients with recurrence underwent subsequent extrafascial hysterectomy. The patient with vaginal squamous cell carcinoma underwent chemotherapy. The other 2 cases of LSIL and 1 case of VaINI were tested for cytology and HPV every six months to one year. One of the patients’ cytology showed ASCUS, and the colposcopy-directed biopsy was performed again. The pathology showed chronic inflammation of the cervical mucosa.The other 2 patients' examinations were negative.In the pre-menopausal group, 33 patients had cytological abnormalities during follow-up, including 22 cases of ASCUS, 2 cases of ASC-H, 7 cases of LSIL, and 2 cases of HSIL. 25 patients underwent colposcopy-directed biopsy. Among the remaining 8 cases,7 patients with cytology suggesting ASCUS and HPV-negative did not undergo colposcopy, and the cytology turned negative after review. In another patient,the cytology showed LSIL and HPV was positive. The patient refused the colposcopy and was treated with vaginal medication. Afterwards, the cytology and HPV turned negative. The results of biopsy pathologic diagnosis were 5 cases of HSIL, 5 cases of LSIL, and 2 cases of VaINII-III. The recurrence rate was 2.34%, which was identified 4–28 months after the primary therapy. 3 patients with recurrence underwent extrafascial hysterectomy, and 2 patients underwent repeated CKC. 5 patients with LSIL underwent cytology and HPV testing every six months to one year. These patients were under observation, and cytology and HPV testing had turned negative.2 cases of VaINII-III patients underwent vaginal lesion resection and vaginal medication. Posterior colposcopy biopsy revealed chronic mucosal inflammation.Only one patient with recurrence had positive margins (Table 7). This patient relapsed after 28 months of follow-up. Because she was young and required to preserve fertility, she underwent secondary CKC. Postoperative pathology showed HSIL and negative margin, and then the patient was reexamined regularly. Follow-up until now, HPV and cytology are all negative.

**Table 7:** Follow-up results of patients before and after menopause

	Post-menopausal patients (n = 120)	Pre-menopausal patients (n = 240)	*χ*^2^	*P*-value
Follow-up, n (%)	104 (86.67%)	213 (88.75%)	0.330	0.566
TCT abnormal, n	17	33		
ASCUS	12	22		
ASC-H	2	2		
LSIL	1	7		
HSIL	1	2		
AGC	1	0		
Pathology, n	14	25		
LSIL	2	5		
HSIL	4	5		
VaIN	1 (VaINI)	2 (VaINII-III)		
Normal	7	13		
Recurrence, n (%)	4 (3.85%)	5 (2.34%)	0.569	0.451
Positive margin of recurrence^c^, n	0	1		
Negative margin of recurrence^c^, n	4	4		

^c^margin status after primary CKC

*AGC* atypical glandular cells, *ASCUS* atypical squamous cell of undetermined significance, *ASC-H* atypical squamous cell-cannot exclude HIS, *HSIL* high-grade squamous intraepithelial lesions, *LSIL* low-grade squamous intraepithelial lesions, *NILM* negative for intraepithelial lesion or malignancy, *SCC* squamous cell carcinoma, *Undone* no ThinPrep cytologic test

## Discussion

Worldwide, cervical cancer is the most common malignant tumor of the female reproductive system. HSIL is a well-defined precursor lesion of cervical invasive squamous cell carcinoma. CSIL often occurs in 25- to 35-year-old women. Recently, it is being found even in a younger age group. The morbidity after menopause decreases by 7%-11% according to the literature [3, 4].However, with the increase in the elderly population, the incidence of cervical cancer in elderly women is also increasing correspondingly. It is usually identified late and has a poor prognosis, which seriously threatens the physical and mental health of elderly women. Therefore, we should pay attention to cervical cancer screening in post-menopausal women. Cervical cancer can be prevented by early detection and proper treatment of HSIL.

### Screening methods in post-menopausal women

Previous data suggest that HSIL is not rare in post-menopausal women. Chen et al. [5] reported that among 1,113 cases of CIN3 4.3% occurred in post-menopausal women. Cheng et al. [6] studied 119 CIN2-3 cases that occurred in post-menopausal women, accounting for 6.5% of the 1,810 cases. Li et al. [7] reported that the infection rate of HPV in post-menopausal women was 9.55%. Asciutto K et al. [8] concluded that postmenopausal women with normal cytology testing positive for HR-HPV mRNA were at increased risk for the development of high-grade cervical intraepithelial neoplasia, in contrast to women with a negative HR-HPV mRNA outcome.Hence, post-menopausal women should undergo regular cervical cancer screening. Cytological analysis and HR-HPV DNA test are the main methods for cervical cancer screening. In our study, there was no significant difference in the consistency between the cytology and biopsy histology and the HR-HPV DNA positive rate before and after menopause. The efficiency of detecting HSIL and higher lesions through cytological analysis and the HR-HPV DNA test does not differ between post- and pre-menopausal women. This indicates that these 2 routine screening methods should be also suitable for post-menopausal women.

### Difficulty of colposcopy in post-menopausal women

Colposcopy is an essential tool in the diagnosis of early cervical disease. It is used for the evaluation of patients with an abnormal cytology and persistent HPV infection. Colposcopy-directed biopsy is the golden standard for the diagnosis of cervical cancer and its precursors [9]. Post-menopausal women usually have declining estrogen levels, atrophy of the cervix, and retraction of the SCJ.Thus, lesions are more often localized in the endocervix. These topographical changes cause a transformation zone that is rarely detected and may lead to a higher incidence of unsatisfactory colposcopy and a decrease in the accuracy of colposcopy. Boulanger et al. [4] reported that CIN was localized in the canal in 44% of cases after menopause, whereas before menopause, the percentage was only 12%. Our results showed that the satisfactory rate of colposcopy was significantly lower in the post- than in the pre-menopausal group. Hence, some methods can be used to improve the satisfaction rate and accuracy of colposcopy. Endocervical curettage should be performed at colposcopy in elderly women. A partial endocervical SCJ is visible using the Kogan Endo Speculum or hygroscopic dilatator [10].Furthermore, Richards et al. [11] reported that the use of vaginal estrogen cream twice per week for 6 weeks for patients with smear abnormalities and a low estrogen status improved the satisfactory colposcopy rate and improved the accuracy of the prediction of true high-grade pre-invasive disease. We can also use cervicoscopy and microcolposcopy as some authors do [12–14].

### Treatment for post-menopausal women with HSIL

The standard of diagnosis and treatment for post-menopausal patients with HSIL has not yet been established at present. Hysterectomy is sometimes selected as the primary treatment for post-menopausal patients with HSIL because they have no fertility requirement but are concerned about the persistence or progression of the disease. However, it is not a standard treatment. It is an extensive procedure, and mortality increases with age. When hysterectomy is performed as a primary mode of treatment for high-grade cervical lesions, the percentage of unexpected invasive cancer is much higher than in cases where conization was done. Vesna Kesic et al. [15] showed that invasive cancer was found in 16.04% of hysterectomy cases and in 3.75% of conization cases. Therefore, hysterectomy is an inadequate treatment in cases of unrecognized invasive cancer for post-menopausal patients. Some authors feel that hysterectomy is excessive even in microinvasive cancer and prefer to perform a conization up to 3 mm. As one type of cervical conization surgery, CKC has been widely performed as a diagnostic and therapeutic procedure for patients with HSIL. CKC should be performed first because the punch biopsy is unable to adequately assess the depth of invasion. CKC can provide more exact pathological information, particularly to evaluate CSIL grading and stromal invasion. In our study, the upgrading between biopsy and conization was significantly higher in the post- than in the pre-menopausal group (11.67% vs. 5.42%). Data show that diagnostic CKC can provide guidance for choosing appropriate surgical procedures following conization treatment for post-menopausal women. If microinvasive or invasive cervical cancer is found after CKC, further adequate treatment can be performed. CKC is also a definitive treatment for patients with HSIL. However, the positive margins are a major problem. Cheng et al. [6] reported that the overall positive margin rate of conization in post-menopausal patients was as high as 20.8%, which was significantly higher than that (10.9%) in pre-menopausal patients. Ghaem-Maghami et al. [16] demonstrated by a meta-analysis that the positive margin rate of conization was higher in post-menopausal patients. Hasegawa et al. [17] showed that the length of the cone removed from the post-menopausal patients was significantly longer than that removed from the pre-menopausal patients. In this study, the positive margin rate of CKC was 25.83% in the post-menopausal group, which was significantly higher than that (12.50%) in the pre-menopausal group. This result is similar to that of previous studies. Meanwhile, consistent with other reports [18–21],this study found that the rate of positive endocervical cone margins was significantly higher in the post-menopausal than in the pre-menopausal group. This is related to the cervical atrophy and upward transformation zone in post-menopausal women. This indicates that the operation in post-menopausal women should be performed cautiously, and the cone depth should be increased if necessary to make a thin and high cone.

### Residual disease after CKC

Several reports identified age as a predictor of residual disease [18–21].Moore et al. [18] reported that the presence or absence of dysplasia in the CKC ectocervical margin, endocervical margin, and endocervical glands was not predictive of residual dysplasia in post-CKC hysterectomy specimens. Increasing age and severity of disease in the cone specimen were the only factors that accurately predicted residual dysplasia. Many reports [22–24] had shown a significantly higher rate of residual disease after a positive cone margin compared to a negative margin. In our study, there was no significant difference in the post- and pre-menopausal groups. Post-menopausal patients with positive cone margins had significantly higher chances of having a residual lesion than those with negative margins, similar to the pre-menopausal group. The rate of residual disease was not significantly different between the post- and pre-menopausal patients with positive margins. Therefore, consistent with some reports, we found that residual disease was associated with the margin status after CKC.

### Recurrence after treatment

Some literature [25–28] reported that incidence of CSIL relapse was 2.5% to 8.5%. One reason some gynecologists prefer to perform hysterectomy without previous conization is the belief that recurrence is less likely after hysterectomy. This is not always true. Bremond found a 3% recurrence rate after conization and a 2.7% recurrence rate after a total hysterectomy. This indicates that hysterectomy cannot reduce HSIL recurrence. In our study, the recurrence rates of HSIL after CKC were 3.85 and 2.34% in post- and pre-menopausal patients, respectively, consistent with that in previously published reports. In contrast, hysterectomy does not prevent vaginal recurrence, which appears in 0.5–1% or more of cases [4].In our study, there was 1 case of vaginal squamous cell carcinoma and 1 case of vaginal intraepithelial neoplasia after hysterectomy in the post-menopausal patients. Consequently, exhaustive long-term follow-up must be considered an integral part of CKC of HSIL after menopause. Follow-up is even required after hysterectomy because the patient is at risk for developing genital intraepithelial neoplasia years later. Ramchandani et al. [29] reported that persistent/recurrent disease was found in 50% of patients with positive endocervical and/or ectocervical margins but only in 15% of those whose margins were negative. Endocervical margin status and severity of neoplasia significantly predicted the occurrence of persistent/recurrent disease after conization. In our study, only 1 case among all recurrent patients had positive margins, considering both post- and pre-menopausal patients. Therefore, we still need to expand the sample size and extend the follow-up time.

## Conclusions

It is important to take some care in choosing the appropriate treatment for HSIL occurring in post-menopausal women. CKC can be performed as a primary procedure for diagnosis and treatment in post-menopausal patients with HSIL. Sufficiently deep excisions are necessary to avoid positive endocervical margins among post-menopausal patients to reduce residual and recurrent postoperative lesions. At the same time, the indications for hysterectomy should be appropriately relaxed. A satisfactory follow-up period is necessary for the CKC treatment of HSIL, even after hysterectomy.

## Data Availability

All data generated or analysed during this study are included in this published article.

## Abbreviations

CKC: Cold-knife conization
HSIL: High-grade squamous intraepithelial lesion
CSIL: Cervical squamous intraepithelial lesion
LSIL: Low-grade squamous intraepithelial lesion
SCJ: Squamocolumnar junction
AGC: Atypical glandular cells
ASC-H: Atypical squamous cell-cannot exclude HIS
ASCUS: Atypical squamous cell of undetermined significance
NILM: Negative for intraepithelial lesion or malignancy
VaIN: Vaginal intraepithelial neoplasia

## References

[1] Ostor AG (1993). Natural history of cervical intraepithelial neoplasia: a critical review. Int J Gynecol Pathol.

[2] Farzaneh F, Faghih N, Hosseini MS (2019). Evaluation of neutrophil-lymphocyte ratio as a prognostic factor in cervical intraepithelial neoplasia recurrence. Asian Pac J Cancer Prev.

[3] Penna CL, Fambrini M, Fallani MG (2005). Laser CO2 conization in postmenopausal age: risk of cervical stenosis and unsatisfactory follow-up. Gynecol Oncol.

[4] Boulanger JC, Gondry J, Verhoest P (2001). Treatment of CIN after menopause. Eur J Obstet Gynecol Reprod Biol.

[5] Chen Y, Lu H, Wan X (2009). Factors associated with positive margins in patients with cervical intraepithelial neoplasia grade 3 and postconization management. Int J Gynaecol Obstet.

[6] Cheng X, Feng Y, Wang X (2013). The effectiveness of conization treatment for post-menopausal women with high-grade cervical intraepithelial neoplasia. Exp Ther Med.

[7] Li Y, Chen X, Qu P (2015). Analysis of cervical cancer screening results in 220 post-menopausal women. Shandong Med J.

[8] Asciutto KC, Borgfeldt C, Forslund O (2020). 14-type HPV mRNA test in triage of HPV DNA-positive postmenopausal women with normal cytology. BMC Cancer.

[9] Wright TC, Massad LS, Dunton CJ (2007). 2006 consensus guidelines for the management of women with cervical intraepithelial neoplasia or adenocarcinoma in situ. Am J Obstet Gynecol.

[10] McCord ML, Stovall TG, Summitt RL (1995). Synthetic hygroscopic cervical dilator use in patients with unsatisfactory colposcopy. Obstet Gynecol.

[11] Richards A, Dalrymple C (2015). Abnormal cervicovaginal cytology, unsatisfactory colposcopy and the use of vaginal estrogen cream: an observational study of clinical outcomes for women in low estrogen states. J Obstet Gynaecol Res.

[12] Valli E, Fabbri G, Centonze C (2013). Cervicoscopy and microcolposcopy in the evaluation of squamo columnar junction and cervical canal in LSIL patients with inadequate or negative colposcopy. Int J Biomed Sci.

[13] Lukic A, Iannaccio S, Di Properzio M, et al. Microcolposcopy in the diagnostic evaluation of abnormal cervical cytology: when and why to do it. Clin Exp Obstet Gynecol. 2009;36(1):26–30. 19400414

[14] Darwish AM, Kamel MA, Zahran K (2019). Office cervicoscopy versus stationary colposcopy in suspicious cervix: a randomized controlled trial. J Midlife Health.

[15] Kesic V, Dokic M, Atanackovic J (2003). Hysterectomy for treatment of CIN. J Low Genit Tract Dis.

[16] Ghaem-Maghami S, Sagi S, Majeed G (2007). Incomplete excision of cervical intraepithelial neoplasia and risk of treatment failure: a meta-analysis. Lancet Oncol.

[17] Hasegawa K, Torii Y, Kato R (2016). The problems of cervical conization for postmenopausal patients. Eur J Gynaecol Oncol.

[18] Moore BC, Higgins RV, Laurent SL (1995). Predictive factors from cold knife conization for residual cervical intraepithelial neoplasia in subsequent hysterectomy. Am J Obstet Gynecol.

[19] Kalogirou D, Antoniou G, Karakitsos P (1997). Predictive factors used to justify hysterectomy after loop conization: increasing age and severity of disease. Eur J Gynaecol Oncol.

[20] Bae HS, Chung YW, Kim T (2013). The appropriate cone depth to avoid endocervical margin involvement is dependent on age and disease severity. Acta Obstet Gynecol Scand.

[21] Shaco-Levy R, Eger G, Dreiher J (2014). Positive margin status in uterine cervix cone specimens is associated with persistent/recurrent high-grade dysplasia. Int J Gynecol Pathol.

[22] Chen J-y, Wang Z-L, Wang Z-Y (2018). The risk factors of residual lesions and recurrence of the high-grade cervical intraepithelial lesions (HSIL) patients with positive-margin after conization. Medicine (Baltimore).

[23] Johnson N, Khalili M, Hirschowitz L (2003). Predicting residual disease after excision for cervical dysplasia. BJOG.

[24] Chang DY, Cheng WF, Torng PL (1996). Prediction of residual neoplasia based on histopathology and margin status of conization specimens. Gynecol Oncol.

[25] Milojkovic M (2002). Residual and recurrent lesions after conization for cervical intraepithelial neoplasia grade 3. Int J Gynaecol Obstet.

[26] Papalia N, Rohla A, Tang S (2020). Defining the short-term disease recurrence after loop electrosurgical excision procedure (LEEP). BMC Womens Health.

[27] Skjeldestad FE, Hagen B, Lie AK (1997). Residual and recurrent disease after laser conization for cervical intraepithelial neoplasia. Obstet Gynecol.

[28] Gardeil F, Barry-Walsh C, Prendiville W (1997). Persistent intraepithelial neoplasia after excision for cervical intraepithelial neoplasia grade III. Obstet Gynecol.

[29] Ramchandani SM, Houck KL, Hernandez E (2007). Predicting persistent/recurrent disease in the cervix after excisional biopsy. MedGenMed.

